# Computer tomography-based assessment of perivascular adipose tissue in patients with abdominal aortic aneurysms

**DOI:** 10.1038/s41598-024-71283-9

**Published:** 2024-09-03

**Authors:** Daniel Ginzburg, Sebastian Nowak, Ulrike Attenberger, Julian Luetkens, Alois Martin Sprinkart, Daniel Kuetting

**Affiliations:** Department of Diagnostic and Interventional Radiology, University Hospital Bonn, Venusberg-Campus 1, 53127 Bonn, Germany

**Keywords:** Tomography, Computed tomography

## Abstract

This retrospective study investigates perivascular adipose tissue (PVAT) alterations in CT as a marker of inflammation in patients with abdominal aortic aneurysms (AAA). 100 abdominal CT scans of patients with abdominal aortic aneurysms and 100 age and sex matched controls without underlying aortic disease were included. Artificial Intelligence (AI) assisted segmentation of the aorta and the surrounding adipose tissue was performed. Adipose tissue density was measured in Hounsfield units (HU) close (2-5mm, HU_close_) and distant (10-12mm, HU_distant_) to the aortic wall. To investigate alterations in adipose tissue density close to the aorta (HU_close_) as a potential marker of inflammation, we calculated the difference HU_Δ_ = HU_close_-HU_distant_ and the fat attenuation ratio HU_ratio_ = HU_close_/HU_distant_ as normalized attenuation measures. These two markers were compared i) inter-individually between AAA patients and controls and ii) intra-individually between the aneurysmal and non-aneurysmal segments in AAA patients. Since most AAAs are generally observed infrarenal, the aneurysmal section of the AAA patients was compared with the infrarenal section of the aorta of the control patients. In inter-individual comparisons, higher HU_Δ_ and a lower HU_ratio_ were observed (aneurysmal: 8.9 ± 5.1 HU vs. control: 6.9 ± 4.8 HU, p-value = 0.006; aneurysmal: 89.8 ± 5.7% vs. control: 92.1 ± 5.5% p-value = 0.004). In intra-individual comparisons, higher HU_Δ_ and lower HU_ratio_ were observed (aneurysmal: 8.9 ± 5.1 HU vs. non-aneurysmal: 5.5 ± 4.1 HU, p-value < 0.001; aneurysmal: 89.8 ± 5.7% vs. non-aneurysmal 93.3 ± 4.9%, p-value < 0.001). The results indicate PVAT density alterations in AAA patients. This motivates further research to establish non-invasive imaging markers for vascular and perivascular inflammation in AAA.

## Introduction

Prevalence of abdominal aortic aneurysms (AAA) increases with age, affecting up to 5% of individuals older than 50 years^1^. While the condition is usually asymptomatic until rupture, AAA can be deadly, with a mortality rate of up to 85% in such cases^2^. Established risk factors for AAA development and progression include smoking, diabetes mellitus, aneurysmal size, and morphology^3^. However, understanding of aneurysmal progression is still limited. Although newer assessments such as biomechanical analyses, functional and molecular imaging, and assessment of circulating biomarkers are promising, they are unlikely to be adopted in practice in the near future^4^. Currently, AAA diameter and growth rate remain the only routinely employed markers of AAA risk estimation, despite their limitations. As a result, all AAA patients require regular follow-ups, although up to 50% of smaller AAA below 4 cm diameter remain stable^3^. Therefore, further non-invasive imaging markers are necessary to allow for discrimination between slow-growing or even stable and progressive AAA. As progression of atherosclerosis is directly associated with vascular wall and perivascular inflammation^5^, the perivascular adipose tissue (PVAT) has been identified as a region of interest for monitoring vascular wall inflammation and atherosclerosis progression^6^. As inflammation also plays a critical role in the progression of AAA, inflammatory processes can be detected in PVAT during AAA development^6–9^. CT-based analysis of PVAT allows for deduction of imaging biomarkers for non-invasive evaluation of perivascular inflammation. These measurements are based on the assumption that inflamed adipose tissue shows higher density values in CT according to the Hounsfield (HU) scale. This assumption is supported by recent histological studies observing alterations of PVAT induced by vascular injury and in turn its pivotal role in regulating vascular remodeling^10^. Thus, a greater difference in density can be measured between adipose tissue adjacent to the inflamed vascular wall and adipose tissue distant to the vessel^11^. Although encouraging results have been reported for PVAT analysis in coronary artery disease^12^, there is currently limited data analyzing PVAT density in patients with AAA. Furthermore, manual assessment of PVAT is time-consuming and lacks reproducibility, which hampers broader application while AI-assisted evaluation of PVAT may facilitate broader application.

The aim of this study was to investigate potential perivascular inflammatory alterations in patients with AAA as a basis for non-invasive imaging biomarkers and to demonstrate that the analyses method can be automated by AI to facilitate further research in this area.

## Material and methods

The study was performed in accordance with the relevant guidelines and regulations including the Declaration of Helsinki. The study specific approval for retrospective studies has been exempted as per the IRB approval number 303/16 (Ethics Committee, University Hospital Bonn). The need for a written informed consent was waived.

### Patient selection and characteristics

For the AAA group, 100 patients receiving CT angiographies for AAA evaluation during a time interval of 4 years (04/2016—04/2020) were retrospectively included in the study. Patients were selected consecutively in accordance to the prevalence of AAA in the general population regarding gender and age^13^.

Exclusion criteria for the AAA group were defined as following:

- Aortic dissection/ruptured aortic aneurysms or contained rupture
- Surgical or interventional treatment of AAA
- Patients with artefacts due to movement during the scan or beam hardening artefacts (e.g. caused by lumbal spondylodesis or intraabominal vascular coils).
- Underlying connective tissue diseases associated with AAA development
- Additional non-aortic aneurysmal diseases.

**Fig.1:**
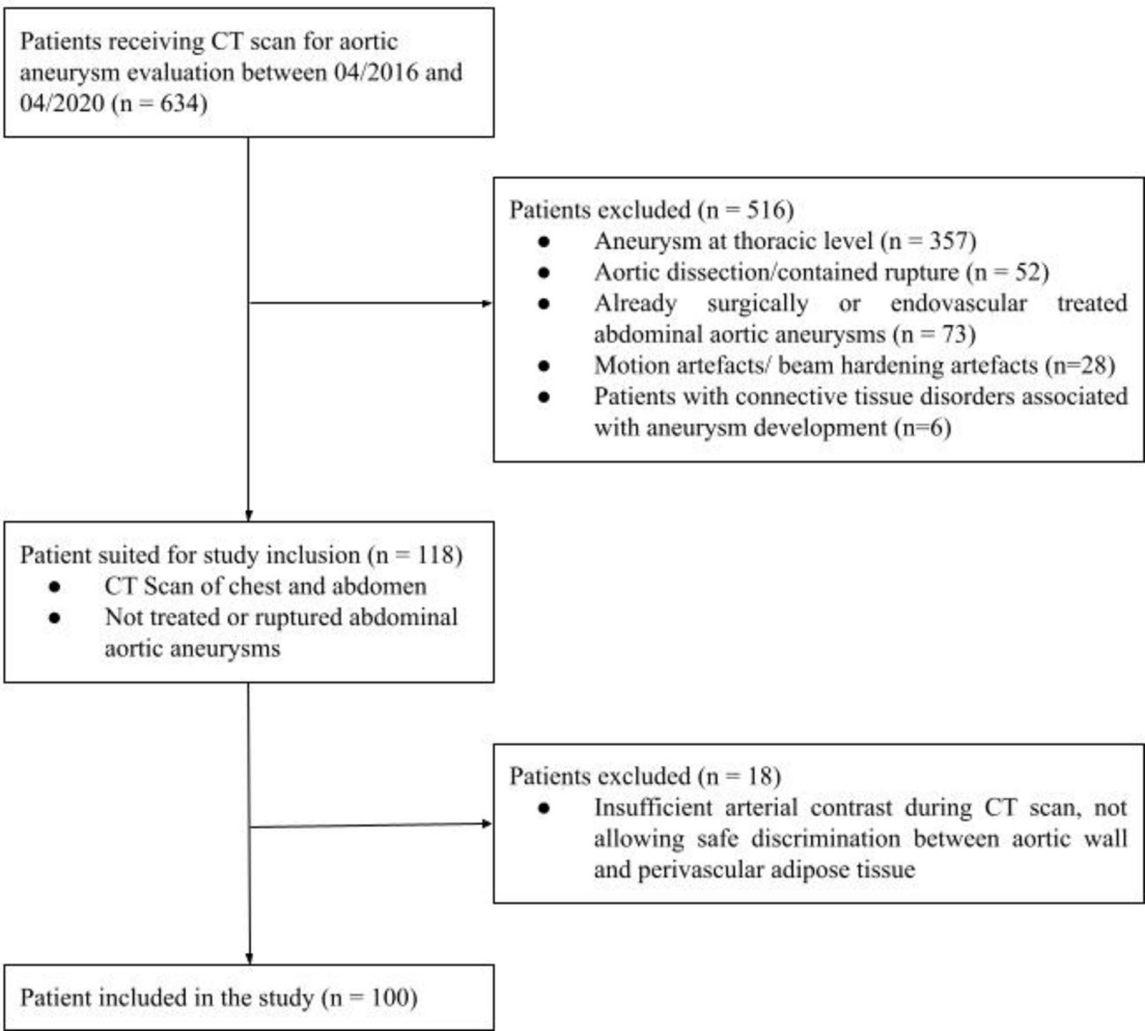
General study outline.

Details regarding the patient selection are listed in the following Fig. 1.

To compile an age- and gender-matched control group, patients without underlying advanced vascular disease who underwent CT scans in the same period as the included AAA patients were considered. Of these, 100 individuals were selected who had only minimal non-calcified plaques and atherosclerotic changes and who had undergone CT scans as part of long-term follow-up for malignancy. Patients with abdominal scan artefacts and with conditions affecting the abdominal aorta or the associated retroperitoneal perivascular fat tissue such as M. Ormond or Takayasu arteritis, were excluded from the control group. Additionally, patients who had received immuno-, chemo-, or radiotherapy were also excluded from the control group due to the possibility of associated inflammatory vascular/perivascular alterations.

### Image acquisition and aortic assessment

Contrast enhanced CT scans were performed on a Somatom Force dual source CT (Siemens Healthineers). Reconstructions of the data set were reformatted in sagittal and coronal planes with a slice thickness of 1 mm with a regular vascular kernel (BV40) and iterative reconstruction (SAFIRE, level 3). Image selection and interpretation was performed with a clinical PACS system viewer (Deep Unity, Dedalus).

The maximal abdominal aortic diameter was evaluated in both the AAA as well as the control group. Aneurysm shapes were subdivided into either saccular or fusiform. The extent of luminal thrombosis (0 cm; < 0,5 cm; 0,5 −1 cm; 1–2 cm; > 2 cm) and the degree of circumferential vasosclerotic changes of the aortic wall (0%; < 25%; 25–50%; 50–75%; 75–100%) were graded in 5 categories (grade 0–4) in AAA patients and controls. For patients with AAA the volume and length of the aneurysmal aortic section was determined.

### Image annotations

**Fig.2:**
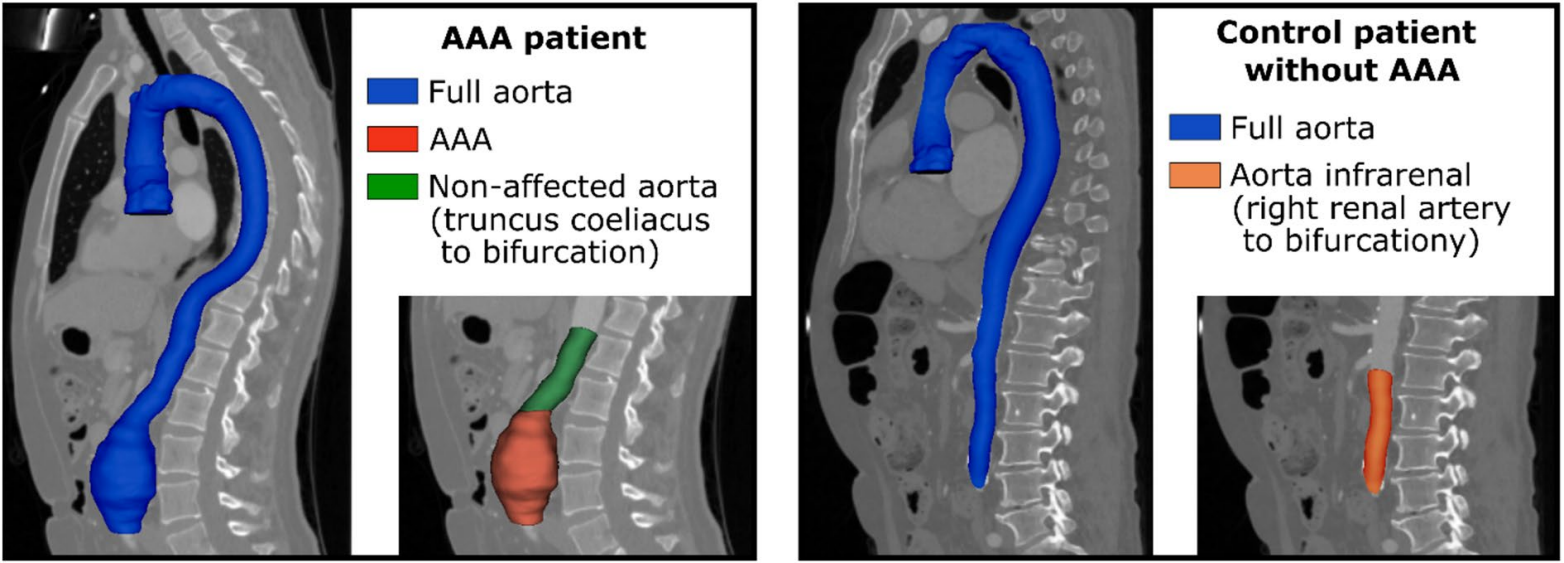
Overview of segmentation and partitioning of the aorta. The aorta was segmented from the right coronary artery to the aortic bifurcation (blue) in all patients. In patients with abdominal aortic aneurysm (AAA), the abdominal aorta was sectioned into the aneurysmal section of the aorta (red) and the non-aneurysmal section of the abdominal aorta between the coeliac trunc and the aortic bifurcation (green). In control patients, the infrarenal section of the aorta was additionally identified (orange).

In a first step, the aorta was segmented from the right coronary artery to the aortic bifurcation. To improve time efficiency of the annotation process, the segmentations were performed iteratively with assistance of artificial intelligence (AI) as described in Supplement S1. The segmented aorta was divided into different sections perpendicular to the central line of the vessel based on several anatomic landmarks placed in in 3D Slicer v4.11.2^14^. For sectioning the aorta of patients with AAA the aortic bifurcation, coeliac trunc, and the inferior and superior ends of the AAA were marked. In control patients, the aortic bifurcation and the junction of the right renal artery were marked to define the infrarenal section of the aorta as seen in Fig. 2.

As most AAAs are observed infrarenally, the aneurysmal section of the AAA patients was compared with the infrarenal section of the aorta of the control patients. To identify voxels surrounding the aorta at different distances from the vessel, the aortic segmentation was iteratively morphologically dilated. An established density-based threshold of −190 HU to −30 HU was subsequently applied for identification of adipose tissue^15^. Detailed information on the landmark based partitioning of the aorta and the extraction of PVAT density is provided in Supplement S2.

### Investigation of the perivascular adipose tissue

Attenuation of the perivascular adipose tissue (PVAT) was measured in HU at two different distances from the aortic wall, namely 2 to 5 mm (referred to as HU_close_) and 10 to 12 mm (referred to as HU_distant_). The area that was closest to the aortic wall including the adventitia, ranging from 0 to 2 mm, was deliberately excluded from the analyses to minimize the potential impact of partial volume effects. The reason for choosing 10–12 mm for HU_distant_ was that fatty tissue should be included at a sufficient distance from the wall to measure an adequate effect, but not too far away to have a close spatial relation to HU_close_. For intra-individual normalization, differences between HU_close_ and HU_distant_ (HU_Δ_) and the fat attenuation ratio HU_ratio_ = HU_close_/HU_distant_ were computed to assess density alterations in PVAT. Both measures were compared in two experiments.

- Inter-individually (AAA vs controls): Assessment of HU_Δ_ and HU_ratio_ in the aneurysmal section of the aorta of AAA patients compared against the infrarenal aortic section of controls.
- Intra-individually (aneurysmal vs non-aneurysmal sections): Assessment of HU_Δ_ and HU_ratio_ in the aneurysmal section of the aorta of AAA patients compared to non-aneurysmal segments inferior of the coeliac trunc to the aortic bifurcation in the same patients.

**Fig.3:**
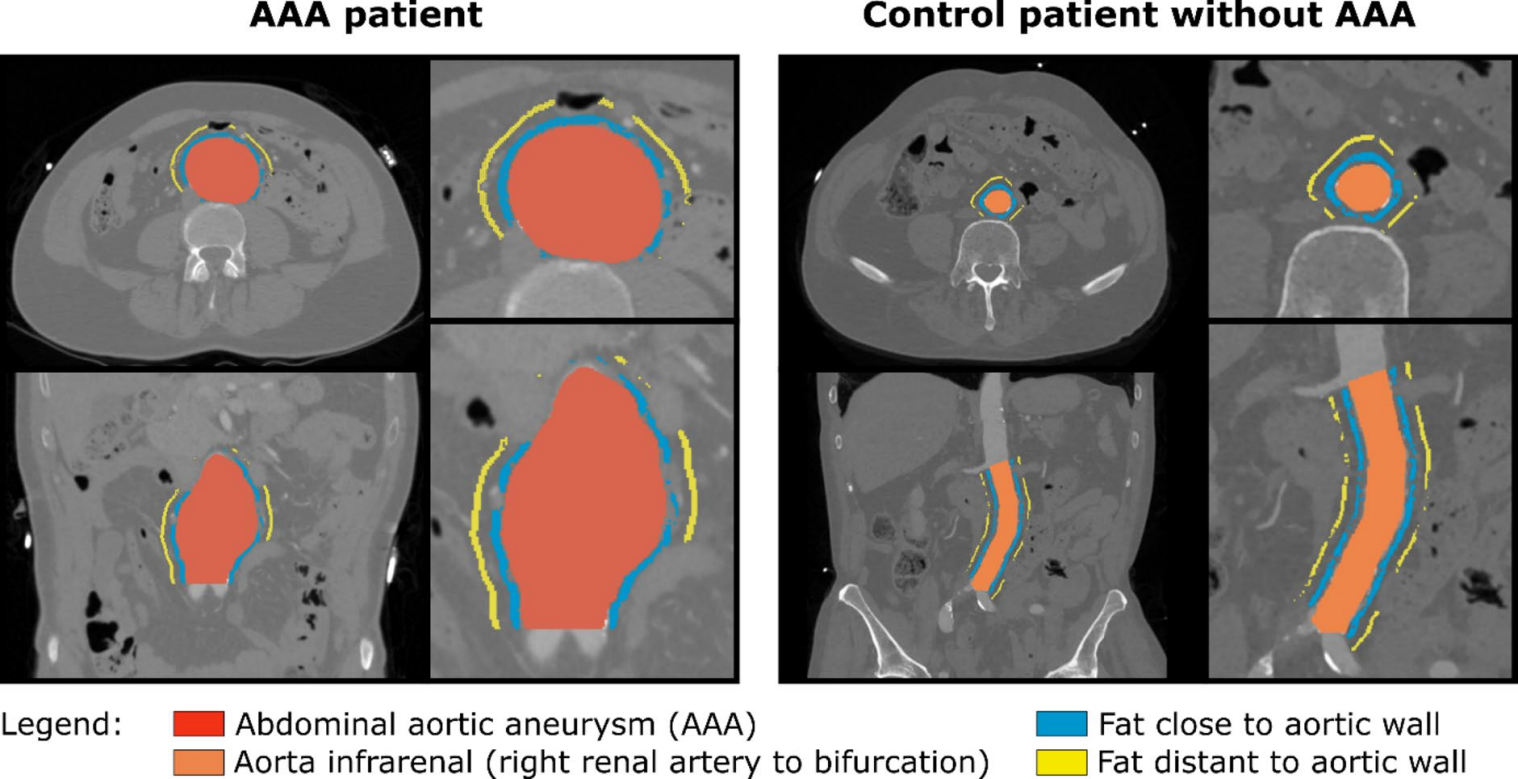
Illustration of the investigated perivascular adipose tissue (PVAT) areas. Mean Hounsfield units (HU) of the PVAT within 2 to 5 mm distance (blue) and within 10 to 12 mm distance (yellow) to the aortic wall were assessed and are referred to as HU_close_ and HU_distant_.

SciPy 1.6.3 was used for statistical analysis^16^. Differences between the groups were assessed by two-tailed t test for independent (i) and dependent (ii) samples. If at least one of the two analysed areas (close or distant PVAT) was smaller than 0.3 cm^3^, the patient was excluded from the respective analysis. Figure 3 illustrates the investigated regions.

### Automation by AI

To assess the potential of AI for automation of future analysis of PVAT in AAA, all annotated images of the study were used to train a (convolutional neural network) CNN for segmentation of the aorta used for centerline extraction and for segmenting aneurysmal sections for localization of AAA. The nnU-Net framework was used to implement a patch-wise generic U-Net with 3D convolutions that divides the input images into patches of size 112 × 112x192 with resolution of 0.72 × 0.72x0.8 mm, which corresponds to a field of view of 80 × 80x153 mm^17,18^. This U-Net was optimized by stochastic gradient descent with Nesterov momentum of 0.99, with a decreasing learning rate starting from 0.01, a batch size of two patches, and oversampling of the foreground voxels. More detailed information on pre-processing, model architecture, and training hyperparameters are presented in Supplement S3.

Annotations from 180 patients (90 with AAA, 90 controls) were used in fivefold cross-validated training. Ensembles of the cross-validated models were evaluated on a hold-out test set (10 with AAA, 10 controls). The segmentation performance was assessed using the Dice score. The mean deviation of predicted and actual inferior and superior ends of the AAA was assessed as performance metric for the localization of the aneurysmal section. For the hold-out test set intraclass correlation coefficients (ICC) were determined for HU_Δ_ and HU_ratio_ calculated by model segmentation and by ground truth annotation. For AAA patients the metrics were determined in aneurysmal section, for the controls in the infrarenal aortic section. ICC was determined in SPSS 27.0.0 with 95% confidence intervals (CI). Also the mean difference per patient between HU_Δ_ and HU_ratio_ calculated by model segmentation and by ground truth is given with CI calculated by bootstrapping with 1000 resamples.

### Code availability

Code for training the U-Net model (https://github.com/MIC-DKFZ/nnUNet) and for evaluation of PVAT based on segmented aortas (https://github.com/ukb-rad-cfqiai/AAA_CT_fat_attenuation_evaluation) is publicly available.

## Results

### Patient characteristics and aortic assessment

Table 1 shows the general patient characteristics. In the AAA group (80% male, 20% female, age: 76.3 ± 9.3 years) the mean of the maximal aortic diameters was 49.8 ± 13.4 mm with 63% of aneurysm showing fusiform configuration, 37% saccular. The mean aneurysmal volume was 120.5 ± 117.6 ml with 25th / 75th quartiles of 49.3 ml / 135.0 ml. The mean aneurysmal length was 116.7 ± 89.4 mm with 25th / 75th quartiles of 59.3 mm / 137.4 mm. In the control group (80% male, 20% female, age: 76.8 ± 9.5 years) the mean of the maximal aortic diameters was 20.7 ± 2.7 mm. The majority of aneurysms showed an extensive degree of thrombosis and non-calcified plaques (75% > 1 cm, 35% > 2 cm max diameter of thrombosis). In the control group the amount of non-calcified plaque was low (90% none, 10% < 0.5 cm diameter). In the AAA group 54% of patients showed calcified atherosclerotic wall alteration affecting more than 50% of circumference, 41% more than 75%. In the control group 71% of patients showed less than 25% circumferential calcified atherosclerotic wall alterations.

**Table 1:** Patient characteristics of the abdominal aortic aneurysm and the control group.

	Abdominal aortic aneurysm	Control
Male	80/100 (80%)	80/100 (80%)
Female	20/100 (20%)	20/100 (20%)
Average age	76.3 ± 9.3 years	76.8 ± 9.5 years
Thrombosis/non-calcified plaque max. diameter*: 0 1 2 3 4	8% 5% 12% 40% 35%	90% 10% 0% 0% 0%
Aortic wall sclerosis percentage^#^ 0 1 2 3 4	7% 20% 19% 13% 41%	16% 55% 15% 11% 3%
Average max. abdominal aortic diameter	49.8 ± 13.4 mm	20.7 ± 2.7 mm
Mean aneurysmal volume	120.5 ml ± 117.6 ml	–
25%/75% quartiles aneurysmal volume	49.3 ml / 135 ml	–
Mean aneurysmal length	116.7 mm ± 89.4 mm	–
25%/75% quartiles aneurysmal length	59.3 mm / 137.4 mm	–

*Thrombosis/non-calcified plaque maximal diameter: 0 = none, 1 =  < 0.5 cm 2 = 0.5–1 cm, 3 = 1–2 cm, 4 ≥ 2 cm.

^**#**^Aortic wall sclerosis percentage: 0 = none, 1 =  < 25%, 2 = 25–50%, 3 = 50–75%, 4 ≥ 75%.

### Assessment of perivascular adipose tissue

**Fig.4:**
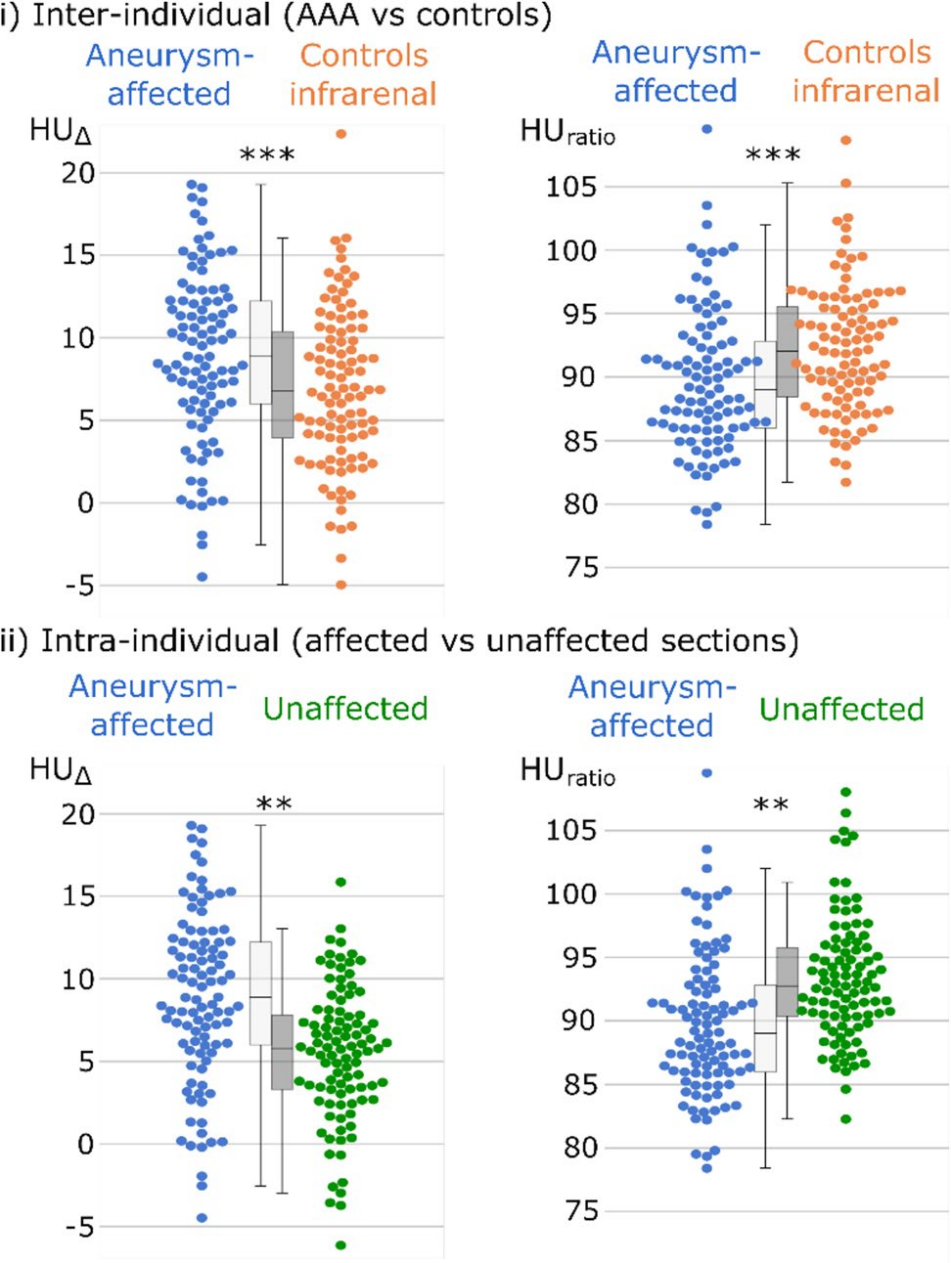
Swarm and boxplots of the investigated perivascular adipose tissues (PVAT). Difference of PVAT attenuation from close and distant measurements (HU_Δ_), along with the perivascular adipose attenuation ratio (HU_ratio_) in % displayed in swarm and box plots. Differences between aneurysm- aneurysmal sections of the aorta (blue) in AAA patients in comparison to the infrarenal section of control patients (orange) were investigated by two-tailed t-test for independent samples. Intra-individual differences between the aneurysmal (blue) and non-aneurysmal aortic sections (green) in AAA patients, were investigated by two-tailed t-test for related samples. **: P-value ≤ 0.01; ***: P-value ≤ 0.001.

Table 2 and Fig. 4 show the results of (i) inter-individual and (ii) intra-individual evaluation of HU_Δ_ and HU_ratio_. For both analyses, one AAA patient was excluded due to very low PVAT volume of less than 0.3 cm^3^ in the aneurysmal segment. Another three patients with AAA were excluded from the intra-individual analysis due to PVAT volumes < 0.3 cm^3^ in the non-aneurysmal segment.

**Table 2:** Inter-individual and intra-individual comparison of PVAT in AAA patients and controls.

Cohort	Investigated section	HU_close_	HU_distant_	HU_Δ_	HU_ratio_
	(i) Inter-individual (AAA vs controls)				
AAA	Aneurysmal segment	−75.0 ± 11.4	−83.9 ± 13.5	8.9 ± 5.1	89.8 ± 5.7
Control	Infrarenal segment	−76.5 ± 10.4	−83.4 ± 12.5	6.9 ± 4.8	92.1 ± 5.5
	P-values	0.337	0.808	**0.006**	**0.004**
	(ii) Intra-individual (aneurysmal vs non-aneurysmal segments)				
AAA	Aneurysmal segment	−75.0 ± 11.4	−83.9 ± 13.5	8.9 ± 5.1	89.8 ± 5.7
AAA	Unaffected segment	−73.0 ± 10.5	−78.5 ± 11.9	5.5 ± 4.1	93.3 ± 4.9
	P-values	** < 0.001**	** < 0.001**	** < 0.001**	** < 0.001**

HU_close:_ mean attenuation of PVAT in Hounsfield units within 2 to 5 mm distance from the aortic wall. HU_distant:_ mean attenuation of PVAT in Hounsfield units within 10 to 12 mm distance from the aortic wall. HU_Δ_: Mean difference of attenuation. HU_ratio_ = HU_close_ / HU_distant_.

Significant values are in bold.

In inter-individual comparisons between patients with AAA and controls (i), higher HU_Δ_ and a lower HU_ratio_ were observed in the aneurysmal segments in comparison to the infrarenal segments of control patients (AAA: 8.9 ± 5.1 HU vs. control: 6.9 ± 4.8 HU, p-value = 0.006; AAA: 89.8 ± 5.7% vs. control: 92.1 ± 5.5% p-value = 0.004).

In intra-individual comparisons patients with AAA (ii) showed higher HU_Δ_ and lower HU_ratio_ in the aneurysmal segments compared to non-aneurysmal segments of controls (aneurysmal: 8.9 ± 5.1 HU vs. non-aneurysmal: 5.5 ± 4.1 HU, p-value < 0.001; aneurysmal: 89.8 ± 5.7% vs. non-aneurysmal: 93.3 ± 4.9% p-value < 0.001).

### Automation by AI

The nnU-Net model segmenting the aorta from the right coronary artery to bifurcation that can be used for automating centreline extraction achieved a mean Dice score of 0.972 ± 0.035 on the 180 patients of the validation sets in fivefold cross-validation and 0.977 ± 0.016 (patient with AAA 0.972 ± 0.021; control patients: 0.983 ± 0.007) on the hold-out test set.

The mean differences per patient between HU_Δ_ and HU_ratio_ calculated based on model segmentation and ground truth annotation evaluated on the test set were low (HU_Δ_ 0.21 [CI 0.01–0.82], HU_ratio_ 0.34% [CI 0.01–1.13%]). ICC analysis also revealed high agreement (HU_Δ_ 0.97 [CI 0.93–0.99], HU_ratio_ 0.96 [CI 0.90–0.98]).The nnU-Net model for locating aneurysmal sections by segmentation achieved a mean deviation between predicted and actual inferior ends of 3.54 ± 4.61 mm and superior ends of 8.22 ± 9.23 mm on the validation sets in fivefold cross-validation. In the hold-out test set mean deviations were 9.01 ± 13.41 mm for inferior and 8.34 ± 7.19 mm for superior ends of AAA.

## Discussion

The main findings of this study are that the analysis of PVAT density, as a surrogate marker of perivascular inflammation, reveals differences in AAA patients not only when compared to a matched control group but also compared with non-diseased segments of the aorta in intra-individual comparison. This indicates the potential of PVAT density analysis as a basis for non-invasive imaging biomarkers in AAA. Furthermore, AI assisted assessment of PVAT density appears feasible, which will facilitate the use and further analysis of respective image-based markers in future large-scale studies.

While aneurysmal diameter and the degree of intraluminal thrombosis are well-established parameters used to predict the risk of AAA growth and related events, the assessment of PVAT as a means to evaluate vascular inflammation in the formation and progression of AAA has scarcely been investigated^15,19^. The perivascular adipose tissue is actively involved in the maintenance of vascular homeostasis^20,21^, and its inflammation has been linked to atherosclerotic changes and hypertension^6^. Additionally, inflammation of the juxta-aortic perivascular fat has been associated with AAA formation^6,8^. A persistent PVAT inflammation can lead to extracellular matrix degradation and vascular wall thinning^8^. While invasive assessment of PVAT is not clinically feasible due to inherent risks of biopsy, CT based analysis allows for non-invasive assessment of PVAT density.

Although PVAT density has been shown to correlate with the degree of perivascular inflammation and coronary atherosclerosis in several studies^11,22^, its analysis for the thoracic aorta has failed to demonstrate any correlation with histopathological findings^23^. The composition of PVAT varies depending on anatomical location, with peripherally increased amounts of white-like adipose tissue compared to the predominantly brown adipose tissue surrounding the thoracic aorta^20,24^. Therefore, PVAT density measurements are expected to vary depending on the anatomical region and to have a variable degree of correlation with inflammatory changes of PVAT. Two CT-based studies examining PVAT of the abdominal aorta have reported increased density values for AAA^15,19^. In fact, Yamaguchi et al. found that PVAT density could serve as a predictor of AAA growth^15^. The present findings provide additional backing for the concept of PVAT alteration adjacent to aneurysmal sections of the aorta, thus requiring further research into PVAT composition as potential predictors of AAA development and progression.

In addition to demonstrating alterations in perivascular adipose tissue density in patients with AAA compared to patients without aortic pathology, similar differences in PVAT density could also be demonstrated by us in intra-individual comparisons of diseased and non-diseased sections of the AAA group. This supports the assumption of inflamed PVAT surrounding AAAs instead of a generally altered perivascular attenuation in patients with aneurysmal disease. The inclusion of age- and sex-matched controls further eliminated other potential biases, such as age-related changes in PVAT density.

In the current study, PVAT density measurements were performed slightly different to those reported in previous studies^15,19^. Partial volume artefacts from intra-aortic iodine, beam hardening and blooming artefacts from mural calcifications can potentially cause an artificial elevation of perivascular attenuation. Absolute attenuation differences found in this as well as previous studies are quite low^15,19,25^. Therefore, also minimal artefacts could potentially significantly influence PVAT analysis. Thus, to reduce artefact interference, the area directly adjacent to the aortic wall was excluded from analysis in our study. Furthermore, instead of the absolute value of PVAT density, we focused on the difference and the ratio between the densities of fat tissue closer to the vessel compared to distant fat tissue as normalized attenuation measures. Another strength of this study is the selection of the control group. The selection process included matching for age and sex, as well as exclusion of patients with potential aortic/periaortic disease.

This is the first study, to our knowledge, investigating AI assisted assessment of aortic PVAT. Incorporating AI in this study provided several benefits, including simplification and abbreviation of aortic annotation in the current cohort and the automation of future analysis. The AI algorithm could help in the standardization and accessibility of PVAT assessment in AAA and enable the transfer of the analysis to potential research collaborators. To ensure practicality for CT images of clinical routine, the aortic segmentation method ideally should allow for assessment of variable scan lengths, including only the thoracic/abdominal aorta or both regions. However, this poses a challenge as a CNN typically requires a fixed input shape. Moreover, segmentations should be performed on high-resolution examinations to allow for precise measurements of PVAT in a region surrounding the aorta of only a few millimetres. To address these challenges a patch-wise 3D CNN was developed with the nnU-Net framework that divides input images into fixed-size patches of high resolution, enabling the analysis of datasets with variable scan lengths. Furthermore, nnU-Net has demonstrated high performance in segmentation challenges, including the aorta^16^.

It is important to note that this is a retrospective, single-center investigation. While the number of patients included is relatively high compared to previous studies investigating PVAT in patients with AAA, the sample size may still be considered low in relation to the absolute attenuation differences observed. Moreover, the patient cohort consisted mainly of individuals with advanced AAA, and follow-up data were not collected due to the fact that the majority of patients underwent endovascular aortic repair treatment, thereby limiting the reliability of follow-up measurements. Lastly while it has been shown that inflammatory changes of the PVAT are a major factor in AAA development it remains difficult to prove that alterations in density of PVAT are only based on inflammation. Therefore, long term follow-up studies are required to prove the prognostic implications of CT based PVAT analysis.

## Conclusion

The results indicate PVAT density alterations in AAA patients that could have potential to provide valuable imaging markers of perivascular inflammatory alterations. This motivates further research to establish non-invasive imaging markers for vascular and perivascular inflammation in AAA for risk stratification regarding aneurysmal growth and rupture risk. Therefore, multicentre studies should be initiated to investigate AI-based PVAT assessment in AAA patients longitudinally.

## Supplementary Information

Supplementary Information.

## Data Availability

The datasets generated during and/or analysed during the current study are available from the corresponding author on reasonable request.
