# Supplementary material for: Computer tomography-based assessment of perivascular adipose tissue in patients with abdominal aortic aneurysms
Source: Sci Rep. 2024 Sep 3;14:20512. doi: 10.1038/s41598-024-71283-9 (PMC11372190; doi:10.1038/s41598-024-71283-9)
Supplement: Supplementary file 1 — Supplementary Information. [file 41598_2024_71283_MOESM1_ESM.docx]

**Supplement**

**S1 AI-assisted annotation for aorta segmentation**

To improve time efficiency of the annotation process and to automate future analyses, the segmentations were performed iteratively with assistance of artificial intelligence (AI). The process involved manual segmentation of the aorta in 20 patients with AAA and in 20 control patients in 3D in 3D Slicer v4.11.2 [1]. This initial segmentation was used to train a Convolutional Neural Network (CNN) using the open-source framework nnU-Net [2]. This first segmentation CNN was then used to generate preliminary segmentations of an additional 20 patients with AAA and 20 control patients, which were then optimized manually in 3D Slicer. The model was then again trained with the 80 available manually optimized segmentations to support annotation of all remaining cases, which were subsequently also manually reviewed and optimized.

**S2 Landmark based division of the aorta into sections and PVAT extraction**

Before partitioning the aorta based on the manually placed landmarks, the segmentation is first pre-processed by morphological closure using a 5x5x5 kernel. Then, all segmented voxels are removed, except for the voxels belonging to the two largest contiguous parts. Afterward, the central line within the vessel is determined. For this purpose, a distance map is created in which the distances to the voxels of the aortic wall in an 80x80x10 mm area are summed up for each voxel within the aorta. Then, layer by layer, the voxel with the largest distance is defined as a point on the central line.

To determine the fat voxels with different distances to the vessel the segmentation of the aorta was morphologically dilated with a 3x3 sized kernel that increased the size of the segmentation voxel by voxel. Then for each distance fat voxels were identified by HU thresholding (-190 HU to -30HU).

The sectioning of the aorta and the associating fat voxels per section is performed perpendicular to this central line. For this purpose, the closest point on the central line is determined for a given landmark and the segmentation is then cut by a plane whose normal vector is determined by the course of the central line at this point.

**S3 Pre-processing, model architecture, and training hyperparameters**

During training of the segmentation Convolutional Neural Networks (CNN), the nnU-Net framework pre-processes the data by resampling the data to a uniform resolution of 0.72x0.72x0.8 mm and by normalizing the image values by globally determining the 0.5th and 99.5th percentile, mean, and standard deviation of all foreground voxels. Then, all image values are clipped to the percentiles, the mean is subtracted and it is divided by the standard deviation. Also, during training, the images are augmented by rotation, scaling, mirroring, brightness/contrast/gamma modification, Gaussian noise, blurring, and low resolution simulation. Detailed information about the pre-processing and data augmentation of the nnU-Net framework can be found in [2].

The model architecture is shown in Figure S1. In this study, the full-resolution 3D version of the generic U-Net class of nnU-Net was used. The CNN analyzes patches of size 112x112x192, which corresponds to a field of view of 80x80x153 mm. To increase convergence speed, the mish activation function was used instead of the nnU-Net default leaky ReLu activation for the model that segments only the section of the aorta affected by the aneurysm [3].

By default, nnU-Net performs training using a stochastic gradient descent optimizer and a Nesterov momentum of 0.99, with a "poly learning rate" decreasing from the initial learning rate of 0.01 to nearly 0, a batch size of two patches, and oversampling of the foreground voxels. The model trained for segmentation of the entire aorta was trained for 500 epochs with 5-fold cross-validation. The model trained for segmentation of aneurysm -affected sections of the aorta was trained for 3000 epochs with 5-fold cross-validation, as it required longer training to fully converge.


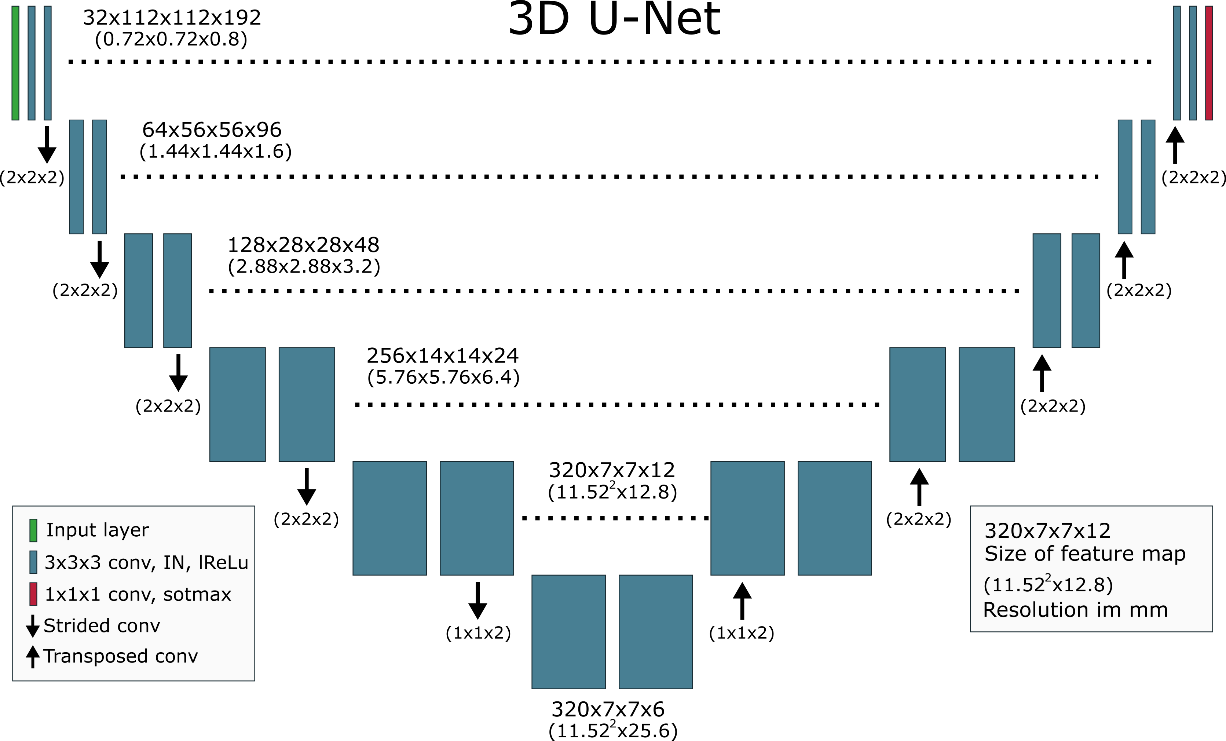
Figure S1: Illustration of the employed U-Net architecture with 3D convolutions (conv) for segmentation of the aorta.

**References**

1. Fedorov, A. *et al.* 3D Slicer as an image computing platform for the Quantitative Imaging Network. *Magn Reson Imaging* **30**, 1323–1341. DOI: 10.1016/j.mri.2012.05.001 (2012).
2. Isensee, F., Jaeger, P. F., Kohl, S. A. A., Petersen, J. & Maier-Hein, K. H. nnU-Net: a self-configuring method for deep learning-based biomedical image segmentation. *Nat Methods* **18**, 203–211. DOI: 10.1038/s41592-020-01008-z (2021).
3. Misra, D. (2019). Mish: A self regularized non-monotonic activation function. *arXiv preprint arXiv:1908.08681* DOI: https://doi.org/10.48550/arXiv.1908.08681 (2019).
